# FCTC Article 2.1 and the next horizon in tobacco policy: Phasing out commercial sales

**DOI:** 10.18332/tid/130673

**Published:** 2020-12-01

**Authors:** Chris Bostic, Marita Hefler, Guy Muller, Mary Assunta

**Affiliations:** 1 Action on Smoking and Health, Washington, United States; 2 Menzies School of Health Research, Charles Darwin University, Darwin, Australia; 3 Dutch Cancer Society, Amsterdam, The Netherlands; 4 Global Centre for Good Governance in Tobacco Control, Bangkok, Thailand

**Keywords:** FCTC, tobacco policy, tobacco sales, tobacco endgame

In the absence of the COVID-19 pandemic, many people in tobacco control worldwide would have been at the Hague, Netherlands, from 9–14 November for the 9th Conference of the Parties (COP9) of the WHO Framework Convention on Tobacco Control (FCTC), advocating for even stronger policies against the tobacco epidemic. The COP has been postponed to 2021, but the pandemic did not stop the global civil society from ‘virtually’ gathering to talk about the FCTC, where it is and where it is going.

On Monday 9 November, Action on Smoking and Health (US), the Menzies School of Health Research (Australia), and Health Funds for a Smoke-free Netherlands, held a panel on the largely overlooked FCTC Article 2.1, which calls on governments to go beyond the minimum obligations specified in the treaty. Some countries and governments have already done this, boldly implementing strategies such as standardized packaging^1^ and flavorings bans^2^.

FCTC is strongly weighted toward demand-reduction policies, but a growing number of governments and non-governmental organizations (NGOs) are looking more closely at supply-side interventions. An excellent case was highlighted on the panel. The Netherlands, host of the next COP, is implementing a plan to significantly reduce the number of tobacco points-of-sale^3^. This policy will reduce child access to tobacco products and exposure to tobacco marketing. Importantly, it also has broad public support.

A global group of individuals and organizations have banded together to encourage such thinking. Two panelists discussed Project Sunset, which aims to phase out the sale of commercial combustible tobacco products as a new ‘floor’ for supply-side policy. Project Sunset begins with a simple question: ‘How is it that the most dangerous consumer product in history is more widely available than many basic life necessities?’.

The effort to phase out tobacco sales is built upon the understanding that the industry, not its customers, are the perpetrators of the tobacco epidemic. Nearly all tobacco users become addicted to nicotine as children. By the time they reach adulthood, when they may be legally capable of consenting to addiction (albeit often with inadequate understanding of the magnitude of the dangers of smoking)^4,5^, it is too late. This is not an accident. The industry has made cigarettes and other products as addictive as possible^6^ and targeted their marketing at young people^7^. The industry knows that youth uptake is essential to replace their dead customers.

Demand reduction is, and will remain, vital to combatting disease and death from tobacco use, but society must also address supply. Over the past 70 years, the tobacco industry has repeatedly promised to recall its products if they are found to be harmful^8^. Other often less dangerous products have been removed from the market, such as asbestos, and leaded paint and petrol. It is time to end tobacco industry exceptionalism. Ending tobacco sales will be an enormous boon to health, wealth, the environment, and human rights. It is past time for governments to not only contemplate phasing out tobacco sales, but also to put in place plans to achieve it. These plans should include cessation support for smokers, and financial transition support for retailers and governments. The state of California has done just this, committing substantial resources to reach their goal. They are succeeding; two cities in California will end tobacco sales as of 1 January 2021.

Most anti-tobacco NGOs have text akin to ‘a world free from tobacco’ in their vision statement. It is time to operationalize that vision.
